# The Molecular Mechanism of Fludioxonil Action Is Different to Osmotic Stress Sensing

**DOI:** 10.3390/jof7050393

**Published:** 2021-05-17

**Authors:** Katharina Bersching, Stefan Jacob

**Affiliations:** Institute for Biotechnology and Drug Research gGmbH (IBWF), Hanns-Dieter-Hüsch-Weg 17, D-55128 Mainz, Germany; bersching@ibwf.de

**Keywords:** fludioxonil, fungicide, mode of action, high osmolarity glycerol (HOG) pathway, *Magnaporthe oryzae*, signal transduction, histidine kinase, *MoHIK1*, *HIK1*, phenylpyrrole

## Abstract

The group III two-component hybrid histidine kinase MoHik1p in the filamentous fungus *Magnaporthe oryzae* is known to be a sensor for external osmotic stress and essential for the fungicidal activity of the phenylpyrrole fludioxonil. The mode of action of fludioxonil has not yet been completely clarified but rather assumed to hyperactivate the high osmolarity glycerol (HOG) signaling pathway. To date, not much is known about the detailed molecular mechanism of how osmotic stress is detected or fungicidal activity is initiated within the HOG pathway. The molecular mechanism of signaling was studied using a mutant strain in which the HisKA signaling domain was modified by an amino acid change of histidine H736 to alanine A736. We found that *MoHik1p^H736A^* is as resistant to fludioxonil but not as sensitive to osmotic stress as the null mutant *∆Mohik1*. H736 is required for fludioxonil action but is not essential for sensing sorbitol stress. Consequently, this report provides evidence of the difference in the molecular mechanism of fludioxonil action and the perception of osmotic stress. This is an excellent basis to understand the successful phenylpyrrole-fungicides’ mode of action better and will give new ideas to decipher cellular signaling mechanisms.

## 1. Introduction

Crop losses in the agricultural sector due to diseases, pests and weeds are approximately assessed to be ranging up to 30% of crop production [1]. A significant number of plant diseases are caused by phytopathogenic fungi, and the most common chemical tools for fungal plant disease control are fungicides. One of the most popular and most successful fungicide classes is phenylpyrrole fungicide. Hardly any cases of field resistance have been reported in almost 30 years of intensive use in agricultural plant protection. That makes the active principle of this fungicide class one of the most interesting molecular mechanisms of action in plant-protection research. The phenylpyrrole fludioxonil (4-(2,2-difluoro-1,3-benzodioxol-4-yl)-1H-pyrrole-3-carbonitrile) is a nonsystemic fungicide and shows a remarkable broad activity across all fungal species (except Oomycetes), particularly against the genera *Botrytis*, *Fusarium*, *Magnaporthe*, *Aspergillus*, *Monilinia* and *Penicillium* [2]. The high efficacy against different molds also makes it suitable for both pre- and post-harvest treatments [3]. However, despite this extraordinary activity and the successful usage as an agricultural fungicide worldwide, the mechanism of fludioxonil action in the HOG pathway has still not been clarified in detail.

So far, fludioxonil is known to hyperactivate the high osmolarity glycerol (HOG) signaling pathway through group III hybrid histidine kinases (HK) [4,5]. It is widely assumed that the fungicidal activity of fludioxonil is down to a disordered signal transduction of osmotic stress because the HOG pathway is responsible for cellular adaptation against environmental changes, such as osmotic imbalance [6,7], but the molecular details are still to be resolved [8,9]. Furthermore, group III HK loss-of-function mutants, such as *∆Mohik1* in *M. oryzae*, are resistant towards phenylpyrroles but osmosensitive [6]. The knowledge of the basic biochemistry of osmotic stress sensing is as limited as phenylpyrrole action. Therefore, it is absolutely necessary to obtain a better understanding of both stress-signaling and the mechanism of action of fludioxonil.

Group III HKs are part of multistep phosphorelay systems (MSP) and therefore are prerequisites for the variability of cellular signal transduction in eukaryotic organisms [10]. These signaling systems enable microorganisms to interact actively with their surrounding environments by modulating their physiological status to maintain cellular homeostasis. The MSP generally consist of three components: (I) A two-component sensor hybrid HK, (II) a phosphotransfer protein and (III) a regulatory response protein [11]. Under unstressed conditions, the sensor HK is autophosphorylated at a conserved histidine residue (His) in a HK domain (HisKA domain). Phosphate is permanently transferred to an aspartate (Asp) in the signal receiver (REC) domain within the same HK. The phosphate is then transferred via a histidine-containing phosphotransfer protein (HPt) to the Asp of the REC domain within the regulatory response protein. That means the phosphate transfer within the MSP occurs from a His to an Asp (in the HK), back to a His (in the histidine-containing phosphotransfer protein), and finally to an Asp (in the regulatory response protein) [12]. One of the major drawbacks of studies concerning HK signaling is the acid instability of phosphorylated His residues, making it challenging to identify, quantify and study phosphorylation states, especially by modern phospho-proteome analysis [13]. Additionally, the lack of commercially available antibodies for detecting phosphorylated His residues makes the quantification extremely difficult [14,15]. Consequently, it is still unknown how His phosphorylation in sensor HKs encrypt or transmit external signals [5]. We generated a mutant strain in the filamentous plant pathogen *Magnaporthe oryzae* in order to shed light on HK sensing and the fungicide action of phenylpyrroles. We modified the cytosolic group III HK MoHik1p (MGG_11174) by replacing the amino acid histidine (H736) exclusively with alanine (A736) in the HisKA domain. The resulting mutant strain *MoHik1p^H736A^* is ideally suited to study the participation and function of the His phosphorylation H736 in the HisKA domain in cellular signaling and fungicide action. Therefore, the current study presents evidence that phosphotransfer at amino acid H736 within the group III HK MoHik1p is involved in fludioxonil activity but is not essential for osmotic stress perception.

## 2. Material and Methods

### 2.1. Strains and Culture Conditions

The *Magnaporthe oryzae* strains used in this study: *M. oryzae* 70–15 (*MoWT*, Fungal Genetics Stock Center) and the loss-of-function mutant *∆Mohik1* [6]. The strains were grown at 26 °C on complete medium (CM). The CM at pH 6.5, 2% agar, contains per liter 1 g casamino acids, 10 g glucose, 2 g peptone, 1 g yeast extract, 50 mL nitrate salt solution (containing per liter: 10.4 g KCl, 30.4 g KH_2_PO_4_, 10.4 g MgSO_4_ × 7 H_2_O, 120 g NaNO_3_) and 1 mL of a trace element solution (containing per liter: 1.7 g CoCl_2_ × 6 H_2_O, 1.6 g CuSO_4_ × 5 H_2_O, 5 g FeSO_4_ × 7 H_2_O, 11 g H_3_BO_3_, 5 g MnCl_2_ × 4 H_2_O, 50 g Na_2_EDTA, 1.5 g Na_2_MoO_4_ × 2 H_2_O, 22 g ZnSO_4_ × 7 H_2_O, pH 6.5 adjusted by 1 M KOH). The MM (pH 6.5) contains per liter: 0.25 mL of a 0.01% biotin solution, 1 g glucose, 50 mL nitrate salt solution, 1 mL of a 1% thiamine dichloride solution and 1 mL of a trace element solution.

All chemicals used were p.a. quality unless stated otherwise.

### 2.2. Identification of and Sequence Analysis in Magnaporthe oryzae

A sequence analysis was performed using a BLAST algorithm (https://blast.ncbi.nlm.nih.gov/Blast.cgi) to identify the putative phosphotransfer site within the HisKA domain of the HK MoHik1p. The homologous group III HKs Bos1p in *Botrytis cinerea*, Nik1p in *Neurospora crassa* and Nik1p in *Candida albicans* were included and the alignment was created using Benchling (Cloud-Based Informatics Platform for Life Sciences R&D | Benchling).

### 2.3. Construction of Vectors for Genetic Manipulation

The mycelia of three-day-old liquid cultures were used for the isolation of DNA of *Magnaporthe oryzae* [7]. The purification was performed using the GeneJET^TM^ Plant Genomic DNA Purification Mini Kit (Thermo Fisher Scientific, Waltham, MA, USA), according to the manual’s user guide. Standard molecular cloning procedures were based on [16].

The exchange of H736A in MoHIK1p (MGG_11174) was generated by using the Gibson assembly cloning method [17] conducted with three fragment inserts. The primer pairs SJ-945/SJ-946 and SJ-947/SJ-948 were used to obtain fragments one and two, so that after their assembly, the “CAC” histidine-encoding nucleotide-triplet was changed to the “GCG” alanine-encoding triplet. Fragment three, encoding the glufosinate-ammonium resistance (modified bialaphos resistance gene, BAR), was amplified with the primers SJ-949/SJ-950. The *Bgl*II/*Pst*I-restricted plasmid *pSJ-basic* was used as a backbone. The resulting plasmid *pMoHIK1^H736A^* was sequenced. NEB^®^ 10-β competent *Escherichia coli* strains (high efficiency) were used for bacterial transformation and the construction of plasmids. The fungal transformation of *M. oryzae* was performed using *Agrobacterium tumefaciens*–mediated transformation [18,19,20], resulting in the mutant strain *MoHik1p^H736A^*. The selection of transformants resistant to glufosinate-ammonium was performed by using 100 µg mL^−1^ of the antibiotic in minimal medium, and the successful replacement within genomic DNA of *MoHik1p^H736A^* was confirmed by sequencing with the primers SJ-1815-SJ-1819.

All oligonucleotides used in this study were generated by Eurofins Genomics (Ebersberg, Germany) and listed in Table 1.

### 2.4. Vegetative Growth Assays

The antifungal activity of fludioxonil and the stress tolerance of the mutant strains were conducted according to [6]. Agar blocks of approximately 0.8 cm diameter from 11-day-old *M. oryzae* (*MoWT*, *∆Mohik1* and *MoHik1p^H736A^*) cultures grown on CM were cut out to be tested and transferred on CM or MM agar plates with different stress-inducing compounds (fludioxonil, sorbitol, KCl, NaCl, H_2_O_2_, NaNO_2_). The cultures were grown for 7 days at 26 °C.

### 2.5. Germination Assay

The germination assay was carried out according to Jacob et al. 2016 [21].

## 3. Results

### 3.1. Manipulation of H736 within the Sensor Histidine Kinase MoHik1p

**Figure 1:**
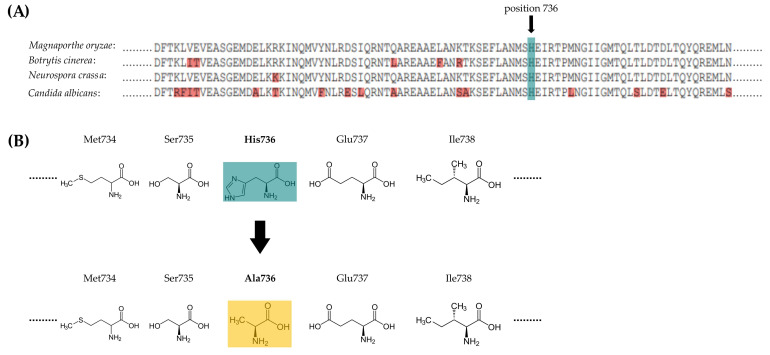
(**A**) Comparison of a protein sequence from the HisKA domain of the group III HK in different fungi. The putative histidine phosphotransfer sites are highlighted in blue. The differences between the sequences are highlighted in red. (**B**) Schematic illustration of the amino acid exchange from histidine (blue) to alanine (yellow).

The HOG signaling pathway is responsible for both osmoregulation and fungicide action. We aimed to manipulate the signaling HisKA domain of MoHik1p to understand how external osmotic changes are perceived and transmitted via the HK MoHik1p in the MSP and to compare osmoregulation with the mechanism of action of phenylpyrrole fungicides. Therefore, we identified the histidine H736 in the HisKA domain by BLAST-assisted comparison of the protein sequences of different group III HK orthologs in fungi (*Botrytis cinerea*, *Candida albicans* and *Neurospora crassa*). The histidine H736 was found to be the putative signaling switch for *Magnaporthe oryzae* (Figure 1a, highlighted in blue).

We generated the mutant strain *MoHik1p^H736A^*, in which the phosphorylation-based signal transfer in the HisKA domain of MoHik1p is impaired by replacing H736 with alanine A736 (Figure 1b) for biological validation and to uncover the riddle that osmoregulation is equal to fungicide action.

### 3.2. Histidine H736 Is a Prerequisite for Fludioxonil Action but Not Essential for Osmosensing

**Figure 2:**
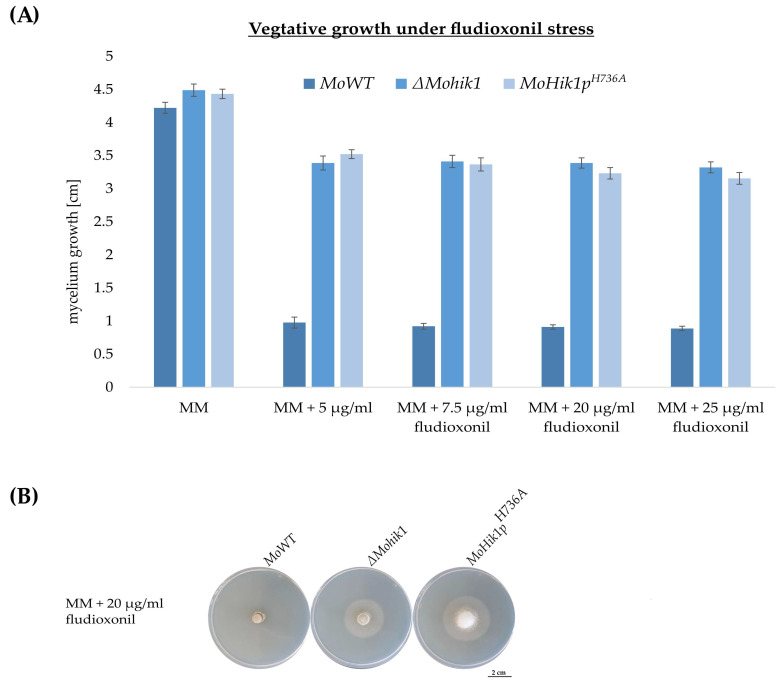
(**A**) Vegetative growth of *Magnaporthe oryzae* wild-type strain (*MoWT*), the loss-of-function mutant *∆Mohik1* and the *MoHik1p^H736A^* mutant under fludioxonil stress. Mycelium growth was measured on MM supplemented with different concentrations of the fungicide fludioxonil. The experiment was conducted for 7 days at 26 °C. (**B**) Pictures of the cultures grown for 7 days on MM + 20 µg/mL.

The loss-of-function mutant *∆Mohik1 is resistant towards the fungicide fludioxonil* [6]. Consequently, *∆Mohik1* is capable of growth even in the presence of high concentrations of fludioxonil up to more than 25 µg/mL of the fungicide (20% growth inhibition as compared to nontreated control), whereas the wild-type strain is strongly sensitive at 5 µg/mL already (80% growth inhibition) (Figure 2). Interestingly, the mutant strain *MoHik1p^H736A^* was found to be as resistant to the fungicide as *∆Mohik1*, although only the histidine H736 is exchanged to A736 in this mutant (Figure 2). This illustrates the fundamental role of H736 in fludioxonil-dependent signal transfer in the HK MoHik1p.

**Figure 3:**
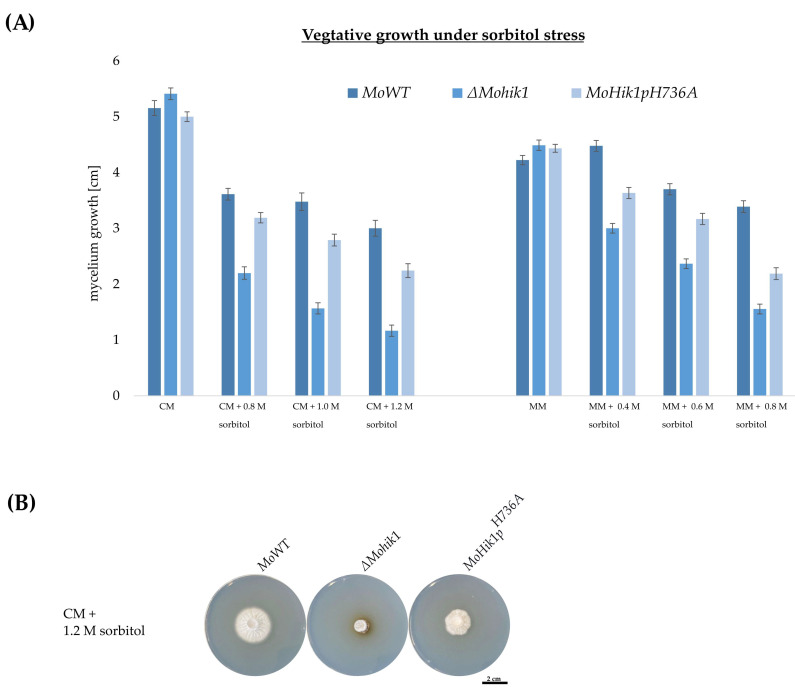
(**A**) Vegetative growth of *MoWT*, the loss-of-function mutant *∆Mohik1* and the *MoHik1p^H736A^* mutant under osmotic stress. The growth was measured on complete media (CM) and minimal media (MM) supplemented with different concentrations of sorbitol. The incubation time was 7 days at 26 °C. (**B**) Pictures of the cultures grown for 7 days on CM + 1.2 M sorbitol.

In contrast to that finding, the results obtained in vegetative growth assays under osmotic stress were different. The loss-of-function mutant *∆Mohik1* is extremely sensitive to osmotic stress compared to the MoWT strain [6]. Thus, *∆Mohik1* is not capable of growing well in the presence of high concentrations of sorbitol (60% growth inhibition), whereas the wild-type strain can do so (29% growth inhibition) (Figure 3a). Interestingly, the mutant strain *MoHik1p^H736A^* was found to be nearly as vital as the MoWT strain under osmotic stress (35% growth inhibition), although the histidine H736, which is so important for fludioxonil signaling, has been removed in this mutant (Figure 3). We present the pictures of the cultures grown on CM medium because of the better visibility of the mycelium (Figure 3b)—the results could be confirmed in MM (Figure 3a, right side). In order to investigate whether the inactivation of H736 affects more than fludioxonil signaling, we monitored growth rates of the *MoWT* and the mutant strains in different media (in accordance with [6]). After 7 days’ incubation on media containing the stress-inducing ingredients, such as KCl and NaCl (salt stress), growth rates were measured. We could not observe any significant differences in vegetative growth between the *∆Mohik1 and* *MoHik1p^H736A^* under salt stress (Table S1).

Both observations, the resistance of *MoHik1p^H736A^* towards fludioxonil and the ability to cope with osmotic sorbitol stress, are consistent with and supported by conidial germination assays (Table S2). Conidia of the mutant strain *MoHik1p^H736A^* were found to germinate under osmotic stress and upon fludioxonil treatment (they are resistant towards fludioxonil). In the end, that means the function of H736 is absolutely required in fludioxonil-dependent signal transfer but not essential for osmoregulation. 

## 4. Discussion

The phenylpyrroles are among the most successful class of fungicides in agriculture. To date, very few cases of field resistance have been reported, despite the intensive commercial use of these fungicides for the past 30 years [22]. Fludioxonil is one of the major representatives of this class of fungicides and is known for acting on the HOG pathway responsible for osmoregulation in fungi [23]. However, the very successful mode of action of fludioxonil has still not been clarified, making this fungicide class and the HOG signaling pathway of high interest for plant-protection research [8]. It is known that fludioxonil action is specific to the group III HK of the HOG pathway and hyperactivates the osmotic MAP kinase cascade without perturbing the cells with the stress of high osmolarity, but the molecular details of how the signal sensing system operates are still missing [5,8,24]. Consequently, detailed knowledge of the mode of action of fludioxonil and its interference with the osmoregulation pathway is of great interest. Accordingly, we studied the role of phosphorylation-triggered signaling of the cytosolic HK MoHik1p in the phytopathogenic rice blast fungus *Magnaporthe oryzae*. We modified the HK in the HisKA domain within MoHik1p (MGG_11174) by replacing the amino acid histidine (H736) with alanine (A736), and the resulting mutant strain *MoHik1p^H736A^* was then used to compare osmotic stress signaling with fungicide action. The mutant strain *MoHik1p^H736A^* was found to be as resistant to the fungicide as *∆Mohik1*, although only the single histidine H736 is exchanged to A736 in this mutant (Figure 2). By contrast, *MoHik1p^H736A^* was able to tolerate osmotic stress nearly as well as the MoWT strain (Figure 3). With these observations, we succeeded in proving that H736 mediates fludioxonil action but is not essential for osmoregulation. Similar findings have already been published for the function of the group III HK CaNik1p from the human pathogen *Candida albicans* heterologously expressed in the model organism *Saccharomyces cerevisiae* [25]. *S. cerevisiae* was transformed with plasmids carrying mutated *CaNIK1* genes, and the resultant transformants were treated with the antifungal fludioxonil. The results indicate that a functional HisKA domain of CaNik1p is essential for the antifungal activity of fludioxonil activating the HOG pathway [25]. In accordance with that, our results clearly illustrate directly in the pathogen itself different molecular mechanisms of signal transduction at MoHik1p and demonstrate that sensing osmotic stress does not completely rely on H736 phosphorylation. That is in contrast to the general assumption that HK signaling is always catalyzed by the ATP-dependent phosphorylation of the His residue in the conserved HisKA domain [26,27,28,29]. Some examples of atypical modes of signal transduction at HKs include the inactivation of HK activity via homo and hetero oligomerization and cross-phosphorylation between HKs. Again, all these modes of signaling require phosphorylation at the His residue in the HisKA domain [30].

Transmembrane HKs generally perceive the signal by assigned periplasmic sensor domains, but cytosolic and membrane-spanning domains also regulate them [31]. The dynamic conformation of the helix bundle upon signal recognition is affected by input domains, such as PAS, GAF and HAMP, which are located at the N-terminal to the HisKA domain, thereby controlling autophosphorylation of the histidine on the HisKA domain [32,33,34]. By contrast, noncanonical HKs without any input domains but exhibiting an additional REC domain between the kinase core and the C-terminal REC domain are common among HKs and can act by binding to small signaling molecules [35].

It is known that osmotic stress and fludioxonil treatment lead to the same result: An activation of the MAPK MoHog1p and, subsequently, the translocation of MoHog1p into the nucleus [8,36]. Furthermore, the major compatible solute produced by *M. oryzae* to cope with high osmolarity and fludioxonil treatment is, in both cases, arabitol [37]. Despite the view that these cellular reactions upon osmotic stress and fungicide action appear to be equal, we demonstrated clearly that the ways of signal perception and transmission are significantly different. Consequently, this report provides evidence of the difference in the molecular mechanism of fludioxonil action and the perception of osmotic stress, despite the impression that the outcome on a cellular level appears to be equal. This is an excellent basis for further research on the successful phenylpyrrole-fungicides’ mode of action and will help to understand molecular mechanisms of eukaryotic signal transduction.

## Figures and Tables

**Table 1:** List of oligonucleotides used in this study.

Name	Sequence (5′→3′)
SJ-945 (HIK1prom-H736-for)	ctggctggtggcaggatatattgtggtgtaaacaaGAAGAAAAGAAGAAAAAGCACCAGGTAATTAATC
SJ-946 (HIK1prom-H736-rev)	gggtgtgcggatttccgcGGACATGTTAGCGAGGAACTCCGAC
SJ-947 (HIK1H736-flank-for)	ttcctcgctaacatgtccgcgGAAATCCGCACACCCATGAACGGTA
SJ-948 (HIK1H736-flank-rev)	agtgctccttcaatatcaTTTGCCCTCTACGTATATCATACTTCTACAGAGGTATATAGTTG
SJ-949 (trpC + BAR-for)	atatacgtagagggcaaaTGATATTGAAGGAGCACTTTTTGGGC
SJ-950 (trpC + BAR-rev)	ctaataaacgctcttttctcttaggtttacctgcaCTAAATCTCGGTGACGGGCAGGACC
SJ-1815 (PCR 1 for H736)	GGGCGATGGAAGGAGATTAC
SJ-1816 (PCR 1 rev H736)	CGCTTAGCCTCAATCTTTGAC
SJ-1817 (PCR 2 for H736)	ACGAGTCGCAAAAGATGTAG
SJ-1818 (PCR 2 rev H736)	CGTCGATAATGGTGAGCAG
SJ-1819 (Seq-primer)	GCTAACCTAATCCGCAGACG

## Data Availability

Not applicable.

## Supplementary Materials

The following are available online at https://www.mdpi.com/article/10.3390/jof7050393/s1, Table S1: Vegetative growth; Table S2: Inhibition of conidial gemination.
